# A Preliminary Examination of Modulation of the LPP by Personal Preference Rankings for Pleasant Experiences

**DOI:** 10.1111/psyp.70443

**Published:** 2026-11-05

**Authors:** Corinne Carlton, Di Yao, Samantha Pegg, Rebecca Mueller, Yinru Long, Lisa Venanzi, Autumn Kujawa

**Affiliations:** ^1^ Vanderbilt University Nashville Tennessee USA; ^2^ Florida State University Tallahassee Florida USA; ^3^ Massachusetts General Hospital/Harvard Medical School Boston Massachusetts USA; ^4^ University of Texas Austin Texas USA

**Keywords:** depression, electroencephalogram, late positive potential, pleasant images, positive emotions, positive valence systems

## Abstract

The late positive potential (LPP) is a robust neural marker of attention toward emotionally salient stimuli, yet it is unknown how the LPP is modulated by personal preferences for specific types of pleasant objects/experiences. The present study aimed to evaluate whether personal preferences for pleasant experiences modulate the LPP to images reflecting those situations. Secondary analyses examined whether depressive symptoms and a positive emotion intervention were associated with individual differences in this modulation. College students (*N* = 92; *M*_age_ = 19.44, SD = 1.15) reported depressive symptoms before randomization to a positive emotion intervention (*n* = 47) or study skills comparison group (*n* = 45). Participants then rank‐ordered a list of eight categories of positive objects/experiences (e.g., cute animals, adventure, social activities, junk food) based on which they enjoyed most and viewed images of each while EEG data were recorded. The LPP was modulated by personal preferences broadly, such that the LPP to images depicting top‐ranked pleasant categories was enhanced compared to low‐ranked pleasant categories; however, significant effects were not observed linearly between each individual ranking. In secondary analyses, depressive symptoms moderated preference effects on the LPP, with greater symptoms associated with less differentiation between highly preferred and less preferred positive stimuli. The positive emotion intervention did not significantly impact LPP modulation by personal preference. Results highlight the utility of considering personal salience in selecting stimuli for clinical neuroscience tasks and indicate that depression may be characterized by difficulty sustaining attention to personally salient pleasant experiences.

## Impact

The present study advances the psychophysiological literature by demonstrating that the late positive potential (LPP) is shaped by idiosyncratic personal relevance. Additionally, by linking individual differences in LPP modulation to depressive symptoms, the study provides meaningful insight into emotional salience processing in those with depressive symptoms. Further, this study highlights important methodological implications for the design of affective electroencephalogram paradigms, suggesting that incorporating personalized stimulus selection may enhance sensitivity to individual differences and psychopathology‐relevant mechanisms.

The late positive potential (LPP) is a positive deflection in the event‐related potential (ERP) waveform that begins as early as 200 milliseconds after stimulus onset and persists throughout stimulus presentation (Hajcak et al. 2010; Schupp et al. 2000). The LPP reflects sustained attention and elaborative processing of motivationally salient stimuli, providing a real‐time, millisecond‐by‐millisecond index of how the brain allocates attentional resources to emotional content (Hajcak et al. 2010; Schupp et al. 2000). The LPP is typically quantified 400 to 1000 milliseconds post‐stimulus onset over central, parietal, and/or occipital electrode sites (Cuthbert et al. 2000; Foti and Hajcak 2008; Hajcak and Dennis 2009) and is reliably enhanced for emotional, including pleasant, images relative to neutral images (McLean et al. 2020; Schupp et al. 2000). Pleasant images such as candy and kittens enhance the LPP in young children as compared to neutral images (McLean et al. 2020), suggesting that even early in development, the LPP is sensitive to pleasant content. Additionally, prior work has shown that more arousing stimuli elicit a more robust LPP as compared to less arousing stimuli (de Rover et al. 2012; Hajcak et al. 2010). Although the LPP is reliably enhanced for pleasant relative to neutral stimuli and increases as a function of emotional arousal (Weinberg and Hajcak 2010), emotional processing is also influenced by factors such as personal meaning and self‐relevance. Stimuli that are personally meaningful or important to an individual may elicit enhanced LPP responses. This raises the question of whether personal preferences for specific types of positive experiences might impact the magnitude of the LPP.

A growing body of work suggests that personal relevance also may influence the LPP. For example, Grasso and Simons (2011) found that children exhibited larger LPP amplitudes when viewing their own parents' faces compared to unfamiliar faces, indicating enhanced sustained attention to personally meaningful social stimuli. Similarly, Tacikowski and Nowicka (2010) reported enhanced LPP activity during processing of self‐related compared to other‐related information, further demonstrating that self‐relevance influences sustained neural processing. Together, these findings suggest that the LPP is sensitive not only to the affective properties of a stimulus but also to its significance for the individual. However, these studies have primarily examined familiarity or self‐relevance, leaving it unclear whether personal preferences for different categories of pleasant experiences similarly modulate the LPP.

Prior research indicates that individuals with depression exhibit altered LPP responses to emotional stimuli across multiple contexts and stimulus types. For example, people with major depressive disorder (MDD) demonstrate blunted LPP responses to both threatening and pleasant stimuli relative to healthy comparisons (Brown et al. 2012; Foti et al. 2010; Hill et al. 2019; Klawohn et al. 2021; Weinberg et al. 2016). This pattern of reduced LPP across emotional valences has been suggested to represent a broad deficit in emotional reactivity and processing in depression. Further, individuals with higher levels of depressive symptoms show smaller modulations of LPP amplitude between pleasant and neutral images, potentially suggesting a reduced capacity to engage with potentially rewarding stimuli at a neural level (Levinson et al. 2019). Taken together, this work shows a clear link between depression and blunted LPP responses. However, prior studies assessing the impact of depression on the LPP have not considered personal preferences for positive stimuli when assessing impact. Indeed, tasks often include a broad variety of pleasant images (e.g., happy faces, cute animals, affiliative images, erotic images) that contain variable personal salience, raising the question about how depression impacts the LPP to different types of stimuli and personal preferences.

Emerging research suggests that the LPP is sensitive not only to the emotional properties of stimuli but can also be modulated by both individual differences in emotional functioning and intentional strategies that enhance positive affect. Altered LPP responses have been observed in depression and other internalizing disorders, suggesting that this neural measure is sensitive to clinically relevant differences in emotional processing (Bress et al. 2023; Buchheim et al. 2018; Kinney et al. 2021). At the same time, savoring and positive up‐regulation strategies have been shown to increase neural responses to pleasant stimuli, as indexed by the LPP (Krompinger et al. 2008; Wilson and MacNamara 2021). Together, these findings suggest that the LPP is sensitive to both individual differences in positive emotion processing and experimental manipulations designed to enhance engagement with positive experiences. Building on this work, Long et al. (2025) developed the Brief Positive Psychological Enhancement (BPPE), a brief single‐session intervention targeting positive valence systems functioning by increasing attention to, engagement with, and reflection on positive experiences. Although BPPE has demonstrated promising effects on positive emotional functioning (Long et al. 2025), it remains unknown whether it influences neural responses to personally preferred pleasant stimuli. Accordingly, the present study examined as a secondary aim whether depressive symptoms and participation in BPPE were associated with individual differences in preference‐related modulation of the LPP.

Despite growing evidence that the LPP is sensitive to pleasant stimuli, a critical gap remains in the literature: it is unclear whether the LPP is specifically modulated by preferences for specific types of positive experiences and whether depressive symptoms or brief interventions targeting positive valence systems impact these responses. As such, the primary aim of the present study was to determine whether personal preferences for specific pleasant experiences modulate the LPP to images depicting these categories. We hypothesized that the LPP would be enhanced for personally preferred positive experiences relative to less preferred positive experiences. A secondary aim was to test whether depressive symptoms and/or participation in a brief positive emotion intervention were associated with a preference‐related modulation of the LPP. We hypothesized that higher depressive symptoms would be associated with a blunted LPP to personally preferred positive stimuli. This hypothesis extends prior findings of reduced LPP responses to broad pleasant stimuli in depression (Foti et al. 2010; Hill et al. 2019; Klawohn et al. 2021; Weinberg et al. 2016) by specifically examining the impact of personal preferences. Additionally, we expected that participants who completed the BPPE intervention would demonstrate a more robust LPP to pleasant images compared to those in the study skills (SS) group, suggesting that interventions targeting positive valence systems may enhance neural responsiveness to positive stimuli. This hypothesis was based on the theoretical mechanism of the BPPE, which increases attention to and engagement with positive experiences (Long et al. 2025), and on evidence that positive valence systems interventions can alter neural responses to rewarding stimuli (Craske et al. 2016; Taylor et al. 2024).

## 1 Method

### 1.1 Participants

A total of 92 undergraduate students (*M*_age_ = 19.44, SD = 1.15) were recruited to participate in the study through a university research sign up system for course credit. Inclusion criteria were undergraduate students between 18 and 24 years of age, and exclusion criteria were hearing or visual impairments that interfered with their ability to complete study procedures.

Most participants identified as women (68.5%, *n* = 63), followed by men (29.3%, *n* = 27), with 1.1% identifying as non‐binary (*n* = 1) and another 1.1% identifying as another gender (*n* = 1). Regarding race and ethnicity, 6.5% identified as Hispanic and/or Latinx, 45.7% as White, 31.5% as Asian, 12% as Black and/or African American, 1.1% as American Indian or Alaska Native, 1.1% as another race, and 8.7% as multiracial. In terms of academic standing, 48.9% were first‐year students; 21.7% were sophomores, 15.2% juniors, and 14.1% seniors.

### 1.2 Procedure

All study procedures were reviewed and approved by the Institutional Review Board at Vanderbilt University. Before study procedures began, informed consent was obtained from all participants. Participants then completed a self‐report battery including a questionnaire to assess depressive symptoms (see *Measures* below). Next, participants were randomized to complete either a session of BPPE (*n* = 47) or a comparison study skills (SS) intervention (*n* = 45). The BPPE intervention was a brief, single‐session program designed to promote positive valence systems functioning by increasing attention to, engagement with, and reflection on positive experiences. Participants completed psychoeducation and experiential exercises focused on attending to positive events and gratitude, recounting and savoring positive experiences, seeking out pleasant experiences and activities, and representation and visualization of future positive events (see Long et al. 2025 for more details). The SS comparison condition was matched for duration and format and focused on academic skills and strategies (e.g., note‐taking, active reading, time management, and using campus resources) without targeting emotional functioning. Both interventions were delivered in a structured format lasting approximately one hour. Additional details regarding intervention procedures and clinical outcomes are reported elsewhere (Long et al. 2025). Next, while study staff prepared participants to complete the EEG, participants completed a ranking of their preferred positive experiences (see more in the Personally Relevant Images (PRI) task section in *Measures*), followed by the EEG task. Participants first completed a training session to ensure they understood task instructions and then completed the computerized PRI task while EEG data were continuously recorded.

### 1.3 Measures

#### 1.3.1 Personally Relevant Images Task

The PRI task is a novel task presented during EEG, as with other tasks eliciting the LPP, the PRI task displays key emotional stimuli (more details below). Before beginning the PRI task, participants were asked to think about a list of eight experiences, people, places, and things in terms of how much they enjoy (or think they would enjoy) each category. Categories were selected to capture a broad range of experiences thought to be of interest to college students and also depended on availability of at least 6 images for each (from validated emotional image databases and stock photos). The categories included sports (e.g., football, lacrosse, soccer, gymnastics, etc.; includes watching and playing), cute animals (e.g., puppies, kittens), nature (e.g., beautiful scenery such as forests, lakes, and mountains), cars (e.g., sports and luxury cars), babies and children (e.g., cute photos and funny interactions with kids), junk food (e.g., desserts, candy, chips, etc.), social activities (e.g., fun times with friends, talking to family), and adventure (e.g., rock climbing, skydiving, rollercoasters). They were then asked to rank each category from 1 (most enjoyable for them) to 8 (least enjoyable for them) and were told that they must assign each of the numbers 1–8 to one category. See Supporting Information for information on mean ranking by category (Table S1).

All neutral image stimuli were obtained from the Open Affective Standardized Image Set (OASIS; Kurdi et al. 2017) and the International Affective Picture System (IAPS; Lang et al. 2005) and were selected based on having the lowest mean arousal and medium valence ratings. Positive image stimuli were first selected from OASIS since these images have demonstrated validity in previous studies (Rozenkrants et al. 2008). To ensure each category had six images, additional images were pulled from Pexels and Pixabay (databases of Creative Commons images) based on their face validity with category descriptions. All of the images were in color. Due to potential copyright sharing limitations, materials are available upon request and materials that can be shared under licensing restrictions will be shared where permitted.^1^ After the task was completed, participants reviewed the 60 images and rated how each image made them feel on valence (1 = *completely happy*; 5 = *completely neutral, neither happy nor unhappy*; 9 = *completely unhappy*) and arousal (1 = *completely excited/aroused*; 9 = *completely calm/unaroused*) using a version of the Self‐Assessment Manikin (Bradley and Lang 1994). Positive images overall were rated as more pleasant (*M*(SD)_pleasant_ = 3.16 (0.73), *M*(SD)_neutral_ = 5.70 (0.94), *t* = −21.34, *p* < 0.001) and more arousing (*M*(SD)_pleasant_ = 4.21 (1.00), *M*(SD)_neutral_ = 6.92 (1.40), *t* = −17.83, *p* < 0.001) than neutral images (See Table S7 in the Supplement).

The PRI task involved passively viewing 48 positive images (six images depicting each of the eight categories that were ranked repeated twice for a total of 12 trials per category) and 12 neutral images (repeated twice for a total of 24 trials; objects like cotton swabs, office supplies, socks) while undergoing continuously recorded EEG. Images were presented for 2000 ms each in a randomized order, followed by a fixation cross for 1000 ms. Participants first completed one block in which all images were presented in random order, followed by a break, and then a second block in which all images were presented again in a random order. Presenting images twice yielded 12 trials per positive category prior to artifact rejection and increased the number of observations contributing to LPP averages while maintaining a feasible task length.

#### 1.3.2 Depressive Symptoms

To assess depressive symptoms, participants completed the Inventory of Depression and Anxiety Symptoms (IDAS; Watson et al. 2007), a 64‐item measure consisting of a broad array of symptom subscales. For the present study, only the IDAS General Depression subscale was used given the study aims and prior work showing the association between depressive symptoms and the LPP to positive images (Foti et al. 2010; Hill et al. 2019; Klawohn et al. 2021; Weinberg et al. 2016). Participants rated how strongly they experienced each symptom in the past 2 weeks using a 5‐point Likert scale ranging from 1 (*Not at all*) to 5 (*Extremely*). The IDAS General Depression subscale showed excellent internal consistency (*ω* = 0.90).

#### 1.3.3 EEG Data Collection and Processing

A 32‐electrode BrainProducts actiCHamp system (Gilching, Germany) was used to collect continuous EEG data. To monitor eye movements and blinks, bipolar facial electrodes were placed approximately 1 cm from the outer corners of each eye, as well as 1 cm above and below the right eye. An additional electrode was attached to the back of the participant's neck for referencing HEO and VEO based on Brain Products bipolar‐to‐auxiliary adapter design. Impedances were reduced to below 30 kΩ. Data were recorded online with reference to a single scalp electrode and later re‐referenced offline to the linked mastoids (TP9 and TP10).

EEG data were processed using BrainVision Analyzer (BrainProducts, Gilching, Germany) and bandpass filtered from 0.01 to 30 Hz. Continuous EEG data were segmented −200 ms before stimulus presentation to 2500 ms following stimulus presentation. Data were corrected for eye movements using Gratton's algorithm (Gratton et al. 1983). When a facial electrode was not available to account for eye movements during ocular correction due to signal or connection problems, eye movements were accounted for using channels FP1 with common reference for vertical eye movements and FT9 with FT10 as reference for horizontal eye movements (Pegg et al. 2024). For participants included in EEG analyses, 45.9% used VEO and HEO for ocular correction, 29.8% used FP1 for VEO or FT9/FT10 for HEO, 21.6% used both FP1 for VEO and FT9/FT10 for HEO, and 2.7% removed HEO from processing due to flat HEO and noisy FT9/FT10. Collapsing across positive images, a 2 (EOG: VEO and HEO used vs. Fp1 and/or FT9/FT10 used) × 2 (Valence: Positive vs. Neutral) mixed‐model repeated‐measures ANOVA was conducted to examine the effects of the choice of electrodes measuring EOG and valence on LPP. The analysis revealed a significant main effect of valence (*F*(1,70) = 112.539, *p* < 0.001, *η* ^2^_*p*_ = 0.617), indicating that LPP was larger for positive (*M* = 3.26, SD = 2.86) compared to neutral (*M* = 0.24, SD = 3.52) images. The main effect of EOG was not significant (*F*(1,70) < 0.001, *p* = 0.99, *η* ^2^_*p*_ < 0.001), indicating that the choice of electrodes for ocular correction did not have a major impact on overall LPP magnitude. The interaction between EOG and valence was also not significant (*F*(1,70) = 3.515, *p* = 0.065, *η* ^2^_*p*_  = 0.048), suggesting that the effect of valence on LPP did not differ between the participants who used VEO and HEO and those who used Fp1 and/or FT9/FT10.

Noisy recordings at single electrode sites were interpolated using spherical spline interpolation in BrainVision Analyzer. Among participants included in EEG analyses who had at least one interpolated channel (*N* = 55 participants had at least one interpolated channel), an average of 2.14 channels was interpolated (range = 1–8). Mastoid interpolation was handled separately from scalp‐channel interpolation when applicable (*N* = 13 participants had interpolated mastoids). Semi‐automatic artifact rejection was used to remove artifacts using the following criteria: maximal allowed voltage step of 50 μV/ms, maximal allowed difference of values in intervals of 175 μV (interval length: 400 ms), minimal allowed amplitude of −200 μV, maximal allowed amplitude of 200 μV, and lowest allowed activity in intervals of 0.5 μV (interval length: 100 ms). Data were also inspected visually to remove any additional artifacts and further segmented by category of stimuli and participant's rank order preference for positive image categories and then baseline corrected −200 ms prior to stimulus presentation.

Mean segment counts per rank after artifact rejection are provided in the Supporting Information (see Table S2) and the mean number of segments across ranks ranged from 11.22–11.45. Split‐half reliability was first examined for each individual preference ranking in the high and low preference conditions. Reliability estimates for the individual ranks did not consistently achieve acceptable internal consistency (*r*s = 0.45–0.64). Because each ranking was represented by a relatively small number of trials and the primary analyses focused on averaged preference conditions, split‐half reliability was also calculated for the averaged high‐preference (Ranks 1–3) and low‐preference (Ranks 6–8) conditions. Reliability improved substantially for the averaged high‐preference condition (*r* = 0.79) and was more modest for the averaged low‐preference condition (*r* = 0.60). These findings support the use of averaged preference conditions while also suggesting greater variability in neural responses across lower‐preference categories. Individual‐rank and averaged‐condition reliability statistics are reported in the Supporting Information (Table S3).

Based on prior research (e.g., Hajcak et al. 2010; Moser et al. 2014; Shafir et al. 2015), as well as the timing and scalp distribution of the data in the overall sample (see Figure 1), the LPP was scored as mean amplitudes 400–1000 ms at a pooling of occipital‐parietal sites (O1, O2, Oz, P3, P4, Pz (Cuthbert et al. 2000; Dickey et al. 2021; Foti and Hajcak 2008; Hajcak and Dennis 2009; Long et al. 2024)) and exported as the average response to each positive image category, each positive image ranking, and neutral images. Participants who did not complete the PRI task (*n* = 4), experienced errors during data collection (*n* = 4), or had excessive noise in mastoids (*n* = 7) and overall EEG data (*n* = 4) when processing were excluded from analyses (*n* = 19) for a final sample of *N* = 73.

**FIGURE 1:**
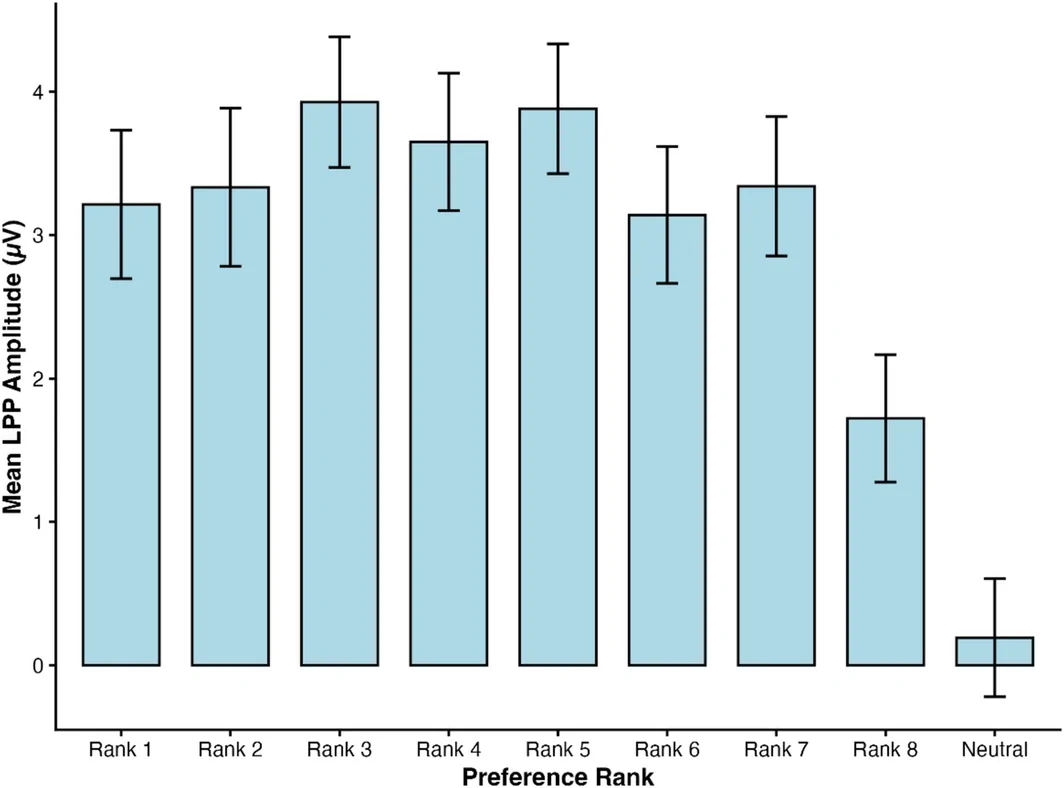
Mean LPP by ranking and neutral condition with standard error bars.

### 1.4 Data Analysis

To assess whether the LPP was impacted by individual preferences for categories of positive stimuli, we first conducted a repeated measures ANOVA across the LPP to all preference rankings and categories 1–8 (see Supplement pages 1–3). Given the complexity in interpreting responses to all 8 categories, we selected ranks 1–3 and 6–8 to maximize differentiation between participants' most and least preferred categories. Inspection of the rank‐order means suggested that the association between preference rank and LPP amplitude was not strictly linear, with middle‐ranked categories showing relatively similar amplitudes. These high (ranks 1–3) versus low (ranks 6–8) preference groups were then used for further secondary analyses of associations with depressive symptoms and randomized intervention group (i.e., BPPE or SS). We conducted ANCOVAs to test the effects of depressive symptoms and the intervention on the LPP, both its overall amplitude and its modulation by preference. These analyses included ranking (high vs. low preference) as the repeated‐measures variable, with depressive symptoms and intervention group as covariates. Although effects of specific positive image categories on the LPP were not the focus of the current study, we also provide information regarding category differences for the mean LPP in the Supplement (see Table S4, Figure S1 and Figure S2).

An a priori *power* analysis was not conducted because these analyses were performed using data from an initial pilot trial designed primarily to evaluate feasibility. A post hoc sensitivity analysis conducted in G*Power 3.1 indicated that, with *α* = 0.05, power = 0.80, and *N* = 73, the study was adequately powered to detect main effects of approximately *f* = 0.13 in repeated‐measures analyses under the assumptions specified. All analyses were conducted in SPSS 28.0.1.1(14).^2^

## 2 Results

### 2.1 Distribution of the Positive Stimuli Ranking

Considerable variability was observed in category preferences across participants (Table S4). These findings demonstrate substantial individual differences in preferences for specific categories of positive experiences. See Supplemental Tables S5 and S6 for descriptives of arousal and valence ratings by categories.

### 2.2 Category‐Level Differences in Subjective Ratings

Repeated‐measures ANOVAs were conducted to examine category‐level differences in subjective ratings (also see Supplement for LPP modulation by image category supplemental analyses). Significant differences emerged across categories for both arousal, *F*(7, 595) = 22.59, *p* < 0.001, *η* ^2^_*p*_ = 0.210, and valence, *F*(7, 595) = 55.98, *p* < 0.001, *η* ^2^_*p*_ = 0.397 where cars, social, and baby categories received the highest arousal ratings (i.e., were rated the least exciting/arousing), whereas cute animals, sports, and adventure categories received the lowest arousal ratings (e.g., were rated as the most arousing).

### 2.3 Modulation of the LPP by Personal Preference Ratings

To examine whether the LPP was modulated by personal preferences, we first conducted a repeated measures ANOVA across the LPP to all preference rankings 1–8 (see Supplement Table S7 for descriptive statistics and descriptions of paired‐samples *t‐*test results). The LPP to neutral images was not included in these analyses as we were specifically interested in the LPP to positive categories and the effect of personal preference rankings on the positive LPP. The LPP was significantly modulated by preference rankings (*F*(7, 504) = 3.43, *p* = 0.001, *η* ^2^_*p*_ = 0.045). However, the LPP did not track participants' stimulus rankings in a strictly linear fashion. See Table 1 for descriptive statistics on the LPP by ranking, and Figure 1 for a visual representation.

**TABLE 1:** Descriptive statistics for LPP by ranking and neutral condition.

Ranking	*M*_μV_	SD_μV_
1	3.21	4.41
2	3.33	4.71
3	3.93	3.88
4	3.65	4.09
5	3.88	3.86
6	3.14	4.07
7	3.34	4.15
8	1.72	3.81
Neutral	0.19	3.52

Abbreviation: LPP, late positive potential.

Given the complexity in interpreting responses to all 8 categories since a non‐linear relation was present, we then collapsed into high (ranks 1–3; *M* = 3.49, SD = 3.53) versus low rankings (ranks 6–8; *M* = 2.73, SD = 3.16). ERP waveforms depicting neutral, low preference averages, and high preference averages and scalp distributions are presented in Figure 2. A repeated measures ANOVA was conducted by entering the LPP to highly preferred images, the LPP to less preferred images, and the LPP to neutral images.^3^ The LPP was significantly modulated by personal preferences (*F*(2, 144) = 50.07, *p* < 0.001, *η* ^2^_*p*_ = 0.411). To interpret this main effect involving three types of stimuli, follow‐up paired‐samples *t*‐tests were conducted comparing LPP responses to high‐preference and low‐preference positive stimuli and to neutral stimuli. LPPs to both high‐ranked (*d* = 1.02, *p* < 0.001) and low‐ranked (*d* = 0.90, *p* < 0.001) pleasant objects/experiences were enhanced compared to neutral, and LPP to high‐ranked was enhanced compared to LPP to low‐ranked (*d* = 0.27, *p* = 0.023; see Table 2). The averages of the LPP to highly preferred images and the LPP to less preferred images were then used for subsequent analyses.

**FIGURE 2:**
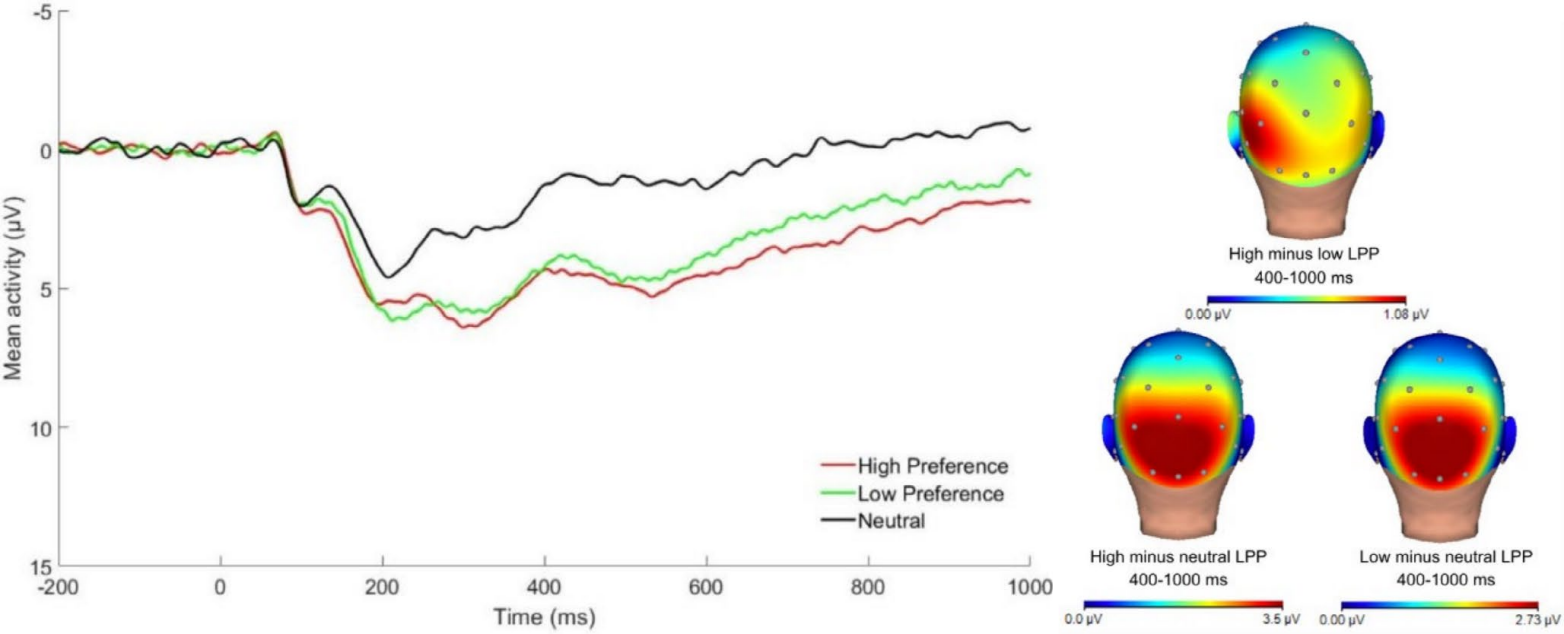
ERP wave for LPP to individual preference rankings and scalp distribution collapsed across neutral, low preference averages, and high preferences averages.

**TABLE 2:** Paired‐samples *t*‐tests examining modulation of the late positive potential (400–1000 ms) by personal preference.

Comparison (condition 1 versus condition 2)	*M* (SD)–condition 1	*M* (SD)–condition 2	Mean diff. 95% CI	*t* (df)	*p*	Cohen's *d* 95% CI
More preferred versus neutral	3.49 (3.53)	0.19 (3.52)	3.30 [2.55, 4.05]	8.75 (72)	< 0.001	1.02 [0.74, 1.31]
Less preferred versus neutral	2.73 (3.16)	0.19 (3.52)	2.54 [1.88, 3.20]	7.67 (72)	< 0.001	0.90 [0.62, 1.17]
More preferred versus less preferred	3.49 (3.53)	2.73 (3.16)	0.76 [0.11, 1.41]	2.33 (72)	0.023	0.27 [0.04, 0.51]

Abbreviations: CI, confidence interval; LPP, late positive potential.

### 2.4 Secondary Analyses of Depressive Symptoms and Intervention Effects

Given that there was evidence that the LPP was modulated by personal preferences, we next conducted secondary analyses examining whether this effect varied as a function of depressive symptoms or intervention assignment. Specifically, repeated measures ANCOVAs were conducted by entering the LPP to highly preferred images and LPP to less preferred images with baseline depressive symptoms or intervention group assignment as a covariate in two separate models.

#### 2.4.1 Main and Interaction Effects of Depressive Symptoms on LPP Modulation by Personal Preference Ratings

Results from the repeated measures ANCOVA demonstrated that the main effect of preference remained significant (*F*(1, 71) = 6.66, *p* = 0.012, *η* ^2^_*p*_ = 0.086), and depressive symptoms significantly interacted with personal preference rankings (*F*(1, 71) = 4.06, *p* = 0.048, *η* ^2^_*p*_ = 0.054). No significant main effect of depressive symptoms on LPP was found (*F*(1, 71) = 0.22, *p* = 0.645, *η* ^2^_*p*_ = 0.003). To facilitate interpretation of the interaction between depressive symptoms and personal preferences, we obtained the LPP preference difference score (*M* = 0.76, SD = 2.78) by subtracting low preference from high preference. The distributions of high (*r* = −0.04, *p* = 0.71) and low preference (*r* = 0.16, *p* = 0.19) LPP responses in relation to depressive symptoms are shown in Figure 3. Greater depressive symptoms were significantly associated with less differentiation in the LPP based on personal preference ratings (*r* = −0.23, *p* = 0.048, see Figure 4 for scatterplot). An outlier was identified in the difference score using Grubbs' test (Grubbs, 1969) and was winsorized by replacing it with the 1st percentile value. When winsorizing that difference score, the correlation no longer reached significance (*r* = −0.23, *p* = 0.054, see Figure S3 in the Supplement). Importantly, the same participant's individual LPP scores for high and low preference LPP responses were not outliers.

**FIGURE 3:**
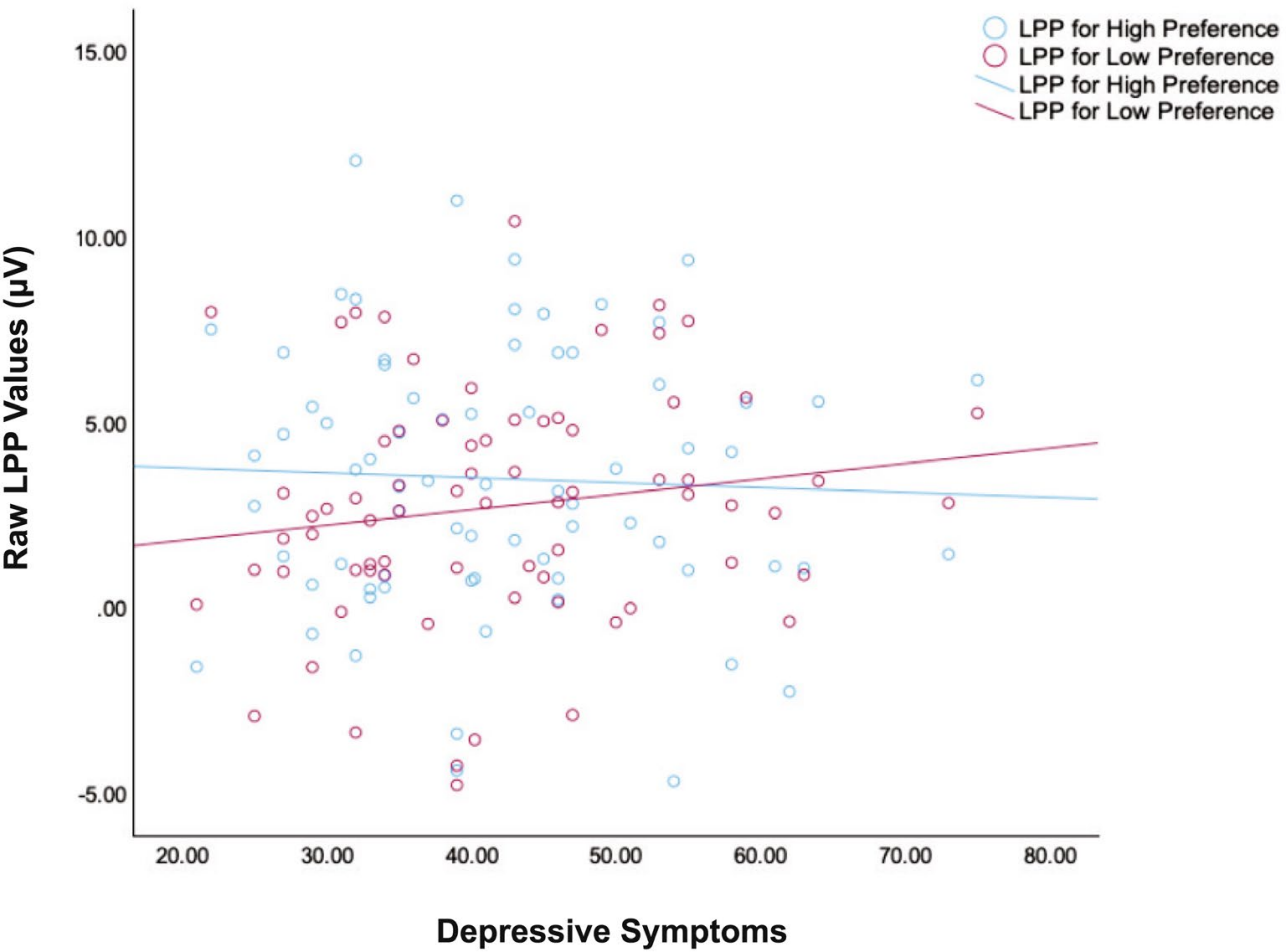
Distributions of high‐ and low‐preference LPP amplitudes in relation to depressive symptoms.

**FIGURE 4:**
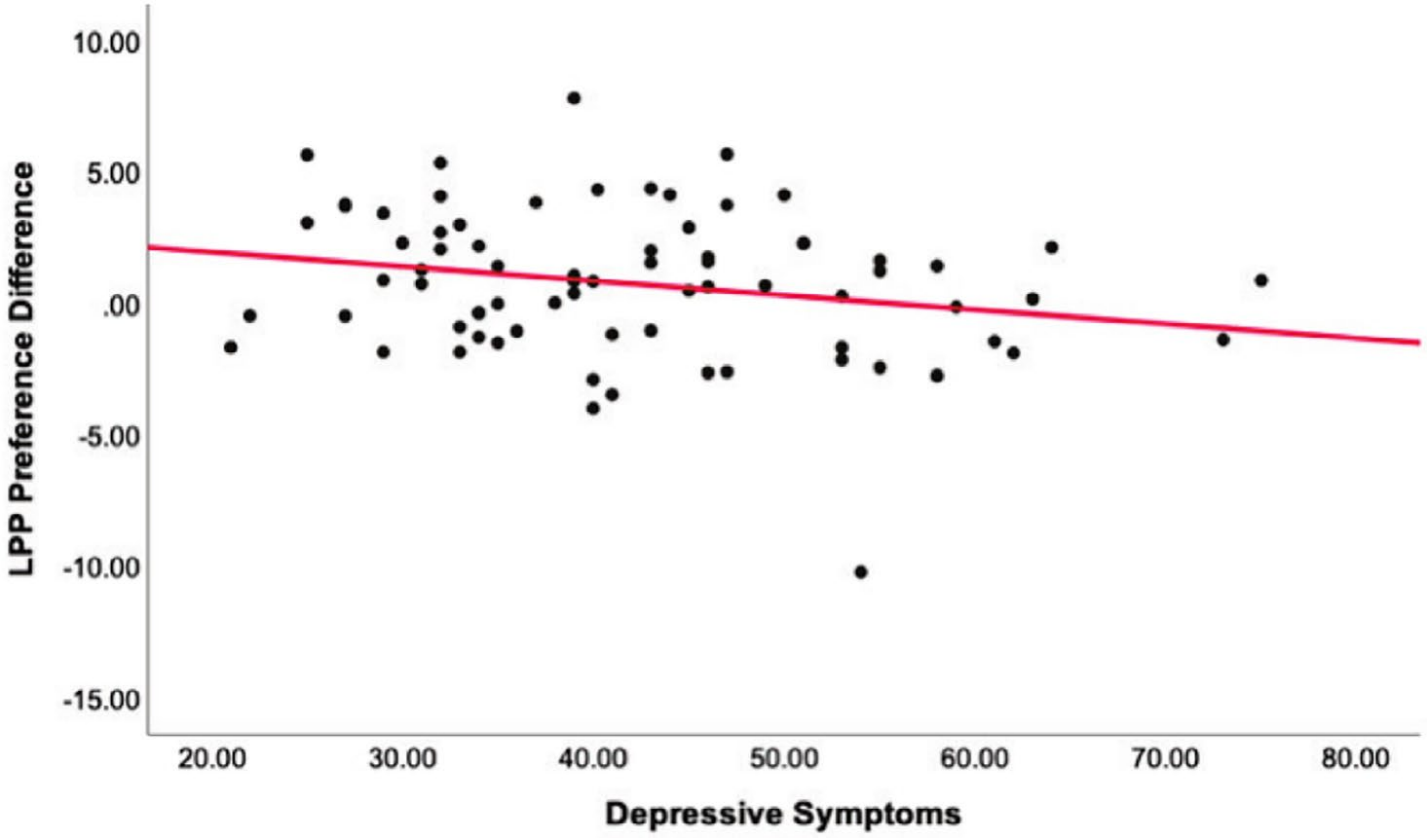
Scatter plot depicting the association between depressive symptoms and the LPP preference difference Score (*r* = −0.23, *p* = 0.048).

To determine whether depressive symptoms were associated with a generalized reduction in neural responsiveness to positive stimuli, we examined associations between depressive symptoms and the positive‐minus‐neutral LPP difference score. Depressive symptoms were not significantly associated with the positive versus neutral contrast, *r* = −0.003, *p* = 0.980. This finding suggests that depressive symptoms were more specifically related to reduced differentiation between highly preferred and less preferred positive stimuli than to generalized blunting of responses to pleasant stimuli. Additionally, supplemental analyses were run regarding the association between depressive symptoms and participants' preference rankings, valence ratings, and arousal ratings (see Supplement page 3).

#### 2.4.2 Main and Interaction Effects of BPPE on LPP Modulation by Personal Preference Ratings

Baseline demographic and clinical characteristics were compared across intervention groups. No significant differences emerged between the BPPE and SS groups on age, gender, race/ethnicity, academic standing, or depressive symptoms (all *p*s > 0.05). Although depressive symptoms were modestly higher in the BPPE group (BPPE: *M* = 44.30, SD = 12.05, range = 21–75; SS: *M* = 39.89, SD = 11.37, range = 22–64), this difference did not reach statistical significance, *t*(90) = −1.81, *p* = 0.07.

No significant group differences (BPPE vs. SS) emerged for the LPP to high (*t*(71) = 0.50, *p* = 0.62) and low ranked (*t*(71) = −0.37, *p* = 0.71) categories, and neutral also did not differ by group (*t*(71) = 0.76, *p* = 0.45). Additionally, no significant differences in average preference rankings, valence ratings, or arousal ratings emerged between the BPPE and SS groups (all *p*s > 0.05).

The repeated measures ANCOVA showed that the main effect of preference remained significant (*F*(1, 71) = 5.74, *p* = 0.019, *η* ^2^_*p*_ = 0.08), and the BPPE intervention did not significantly change the LPP by personal preference ratings (*F*(1, 71) = 1.13, *p* = 0.29, *η* ^2^_*p*_ = 0.021). No significant main effect of BPPE intervention on LPP was found (*F*(1, 71) = 0.01, *p* = 0.921, *η* ^2^_*p*_ = 0.003).

## 3 Discussion

The primary contribution of the present study is the novel evidence that the LPP is modulated by personal preferences for categories of pleasant experiences, which is an important extension of prior research demonstrating that the LPP is sensitive to emotional images using a broad range of categories. Specifically, we found that participants exhibited enhanced LPP amplitude when viewing images depicting stimuli that aligned with pleasant categories they ranked most highly as compared to their personal lowest‐ranked preferences. This finding advances our understanding of the LPP by demonstrating that neural responses to emotional stimuli are not solely determined by generic emotional content, but they are also impacted by individual differences in what categories of positive content that each person finds personally rewarding or meaningful. Indeed, in the present study significant variability in category preference ratings was present among positive stimuli category ratings, further illustrating the need to consider this in future designs leveraging positive emotional stimuli. These results align with theoretical frameworks emphasizing the role of personal relevance and self‐referential processing in amplifying neural responses to emotional stimuli (Schindler et al. 2019; Viskontas et al. 2009).

Our findings that personal preferences for categories of positive stimuli (e.g., babies, cute animals) modulate the LPP have important implications for understanding individual differences in emotional processing. Previous research has established that the LPP is enhanced for personally familiar faces compared to strangers (Grasso and Simons 2011) and for self‐relevant information (Tacikowski and Nowicka 2010) and emotional words embedded in personally meaningful contexts (Schindler et al. 2019). The current study extends this work by demonstrating that highly ranked individual preferences for categories of pleasant experiences also modulate sustained neural attention as compared to lower ranked individual preferences for categories of pleasant experiences. This suggests that the brain's allocation of attentional resources to emotional information may be guided not only by stimulus properties such as valence and arousal, but also by the match between stimulus content and an individual's unique reward hierarchy. This neural response pattern to individualized preferences for categories of positive stimuli may reflect the involvement of key reward circuits in the brain, which have been implicated in processing both emotional intensity and personal relevance (Fossati 2012; Moran et al. 2013). However, it should be noted that the LPP did not track participants' stimulus rankings in a strictly linear fashion and appeared to be disproportionately influenced by the least interesting category. This pattern suggests that the image content selected for the present study may not have fully reflected the range of positive experiences for individuals.

Additionally, category‐level differences in affective properties likely contributed to variability in LPP amplitude, which is a limitation of the current study. Although subjective ratings were collected in the present sample, positive stimuli were drawn from multiple sources, including non‐normed image databases, limiting our ability to fully disentangle the effects of personal preference from category‐level differences in valence and arousal.

Secondary analyses examined whether the preference‐related modulation was associated with depressive symptoms. Results suggest that depressive symptoms significantly impacted the degree to which the LPP was modulated by personal preferences to categories of positive stimuli. Specifically, we demonstrated that individuals with higher levels of depressive symptoms showed less differentiation in LPP amplitude between highly preferred and less preferred categories of positive stimuli. This aberrant processing may reflect a broader pattern of reward processing abnormalities characteristic of depression (Der‐Avakian and Markou 2012). The present findings should also be considered in relation to prior work examining self‐relevant emotional processing in depression. Benau et al. (2019) reported enhanced LPP responses to negative self‐relevant verbal information among individuals with elevated depressive symptoms. Although the current findings involve positive visual stimuli rather than self‐referential verbal material, the two studies may reflect complementary alterations in emotional processing. Notably, depressive symptoms were associated with the high‐minus‐low preference difference score rather than with responses to highly preferred or less preferred positive images individually. This pattern suggests that depressive symptoms may be associated with reduced neural differentiation based on personal preference, rather than a generalized reduction in responsiveness to positive stimuli. However, because this association was sensitive to outlier handling, it should be interpreted cautiously until replicated in larger samples.

Additionally, we assessed whether participation in a positive emotion‐focused intervention (BPPE) was associated with LPP modulation by personal preference to positive images. Contrary to our hypotheses, the BPPE intervention did not significantly enhance LPP modulation by personal preferences to categories of positive stimuli as compared to those assigned to the SS group. This null finding raises important questions about the mechanisms and dosage required for interventions to produce measurable changes in neural indices of emotional reactivity. Although behavioral activation and positive emotion interventions have demonstrated efficacy in reducing depressive symptoms (Craske et al. 2019; Dimidjian et al. 2011; Long et al. 2025; Martell et al. 2021), translating these symptom improvements to changes in underlying neural markers may require more intensive or prolonged intervention.

The current findings have several important implications. First, our results highlight the value of incorporating assessments of personal preferences of categories of positive stimuli into clinical neuroscience research. Standard emotional processing tasks typically employ the same stimuli across all participants, yet our findings demonstrate that individual differences in what people find rewarding significantly influence neural responses. This suggests that “one‐size‐fits‐all” approaches to measuring emotional reactivity may miss important individual variation that is clinically meaningful. Incorporating personalized stimuli or tasks that account for individual preferences may enhance the sensitivity of neural measures for detecting emotion processing deficits and tracking treatment‐related changes. Secondarily, our findings underscore the potential importance of helping individuals experiencing depressive symptoms to identify and prioritize personally meaningful rewarding activities. An additional strength of the present study is that robust LPP responses were elicited using non‐erotic pleasant stimuli. Many investigations of positive emotional processing rely heavily on erotic images because of their reliably high arousal value (e.g., Minnix et al. 2013; Schupp et al. 2000). In contrast, the present paradigm focused on positive experiences that are more representative of everyday life. These present findings demonstrate that meaningful modulation of the LPP can be observed using ecologically relevant positive stimuli and support the utility of such stimuli for studying positive valence systems and affective dysfunction in clinical populations.

Several limitations of the current study should be noted. Our sample consisted of unselected college students, which may limit generalizability to individuals with more severe depression or clinical samples. Additionally, personal preferences were assessed through self‐report rankings, which, while ecologically valid and representing personal salience, may not capture all nuances of individual emotional reactivity and reward sensitivities. Moreover, in the present study we selected categories based on student input on what would be most of interest and feasibly obtained, and as such, may have missed other important categories of positive experiences. For example, we included the category of cars based on student feedback; however, this category was not commonly endorsed as highly ranked and may represent low salience for many individuals. The brief nature of the BPPE intervention and the single‐session design also limited our ability to detect potential neural changes that might emerge with more sustained intervention, and to assess a true “pretreatment versus posttreatment” design. An important limitation is that although participants provided valence and arousal ratings for all images, not all stimuli were drawn from databases with established normative ratings or information regarding image complexity. Although we collected subjective valence and arousal ratings in the present sample, these ratings likely reflect both inherent stimulus characteristics and participants' personal preferences. In addition, other stimulus properties that may influence the LPP, such as visual complexity, were not measured or equated across categories. As a result, it is difficult to fully disentangle the influence of personal preference from category‐level differences in inherent emotional salience. An additional limitation is the reliability of the LPP measures across preference rankings. Individual preference rankings did not reach acceptable split‐half reliability, likely reflecting the relatively small number of trials contributing to each individual ranking score. Reliability improved when rankings were averaged into the high‐ and low‐preference conditions used in the primary analyses; however, the averaged low‐preference condition demonstrated questionable reliability, suggesting that neural responses to lower‐ranked categories remained relatively heterogeneous across participants. This interpretation is consistent with the non‐linear pattern of LPP amplitudes observed across the eight preference rankings. Lastly, there may be interactions between bottom‐up processing of images and personal preferences that impact LPP modulation that we could not account for. For example, the personal level of arousal of each image might interact with personal salience, as well as other factors like complexity or luminosity of images that could impact LPP magnitude.

Future research should expand the range of positive categories available for participants to rank and incorporate behavioral measures of reward learning or effort‐based decision‐making to provide convergent assessments of individual reward hierarchies. Direct comparisons of personally relevant positive and negative stimuli may also clarify whether preference‐related LPP modulation is specific to positive experiences or reflects broader effects of personal relevance. Further, replication of secondary findings presented here in individuals with clinical depression will be important for determining whether reduced preference‐related differentiation extends to more severe cases, while longitudinal studies of more intensive positive emotion interventions could clarify whether these neural responses are modifiable with treatment. Additionally, future work should also aim to better distinguish personal preference from image characteristics. In the present study, valence and arousal differed across categories, but these ratings may themselves reflect participants' preferences and personal associations. Using images matched on valence, arousal, and visual complexity, increasing the number of stimuli per category, and refining the operationalization of lower‐preference stimuli would allow stronger inferences about the unique contribution of personal preference and improve measurement precision. Lastly, future work may benefit from examining the LPP in later time windows (e.g., 1000–2000 ms) during the PRI task to assess more top‐down modulation of the LPP that is present in later time windows (e.g., Bauer 2023; Dillon et al. 2006; Hajcak et al. 2007).

## 4 Conclusion

Taken together, the findings from the present study indicate that individuals process various categories of pleasant images differently based on their personal preference for those categories. Thus, it cannot be assumed that presenting the same broad category of pleasant images would have uniform effects for each person. This is important, as most standardized EEG task designs do not incorporate or control for personal preferences in the selection of categories of images—potentially leading to unexplored differences in neural processing, or interpretation of findings as uniform when really, these processes are moderated by personal preferences for specific positive categories. Additionally, the present study demonstrated that individuals with higher levels of depressive symptoms showed less differentiation in LPP amplitude between highly preferred and less preferred categories of positive images, rather than overall blunting. This suggests that those with higher depressive symptoms may demonstrate an impaired ability to use attentional allocation to positive experiences that are likely to be most enjoyable, which may impact treatment outcomes.

## Author Contributions

**Corinne Carlton:** writing – original draft, methodology, formal analysis, writing – review and editing. **Di Yao:** writing – original draft, formal analysis, writing – review and editing. **Samantha Pegg:** conceptualization, methodology, writing – review and editing. **Rebecca Mueller:** conceptualization, methodology, writing – review and editing. **Yinru Long:** conceptualization, writing – review and editing. **Lisa Venanzi:** conceptualization, writing – review and editing. **Autumn Kujawa:** conceptualization, methodology, writing – review and editing, supervision.

## Funding

This study was supported in part by UL1 TR000445 from the National Center for Advancing Translational Sciences (NCATS) and the National Institutes of Health (NIH). C.C. was supported by the National Institute of Mental Health (NIMH) T32MH018921 and F32MH137993 and S.P. was supported by NIMH F31MH127817.

## Ethics Statement

Ethics approval for this study was obtained from the Vanderbilt University Institutional Review Board through protocol number 200416.

## Consent

Prior to participation, all participants provided informed consent in line with the protocols set by the Vanderbilt University Institutional Review Board and the Declaration of Helsinki.

## Conflicts of Interest

The authors declare no conflicts of interest.

## Supporting information


**Table S1:** Mean ranking by category and distribution of the positive stimuli ranking % placed in the top 3 categories.
**Table S2:** LPP segment counts at Pz by ranking.
**Table S3:** Split‐half reliability by high and low rankings.
**Table S4:** Descriptive statistics for LPP for each positive image category and neutral images sorted from largest to smallest.
**Table S5:** Mean arousal ratings by category.
**Table S6:** Mean valence ratings by category.
**Table S7:** Paired‐samples *t*‐tests examining the difference between pleasant images and neutral image on valence and arousal ratings.
**Table S8:** Image labels and corresponding database.
**Figure S1:** ERP waveforms for LPP to individual categories.
**Figure S2:** ERP waveforms for LPP to individual preference ratings.
**Figure S3:** Scatter plot depicting the association between depressive symptoms and the LPP preference difference score after winsorizing the outlier value (*r* = −0.23, *p* = 0.054).
**Figure S4:** ERP Wave for the Late LPP to Individual Preference Rankings and Scalp Distribution Collapsed Across Neutral, Low Preference Averages, and High Preferences Averages.

## Data Availability

The data that support the findings of this study are available from the corresponding author upon reasonable request.

## Endnotes

^1^
Image identifier labels and sources are included in the Supplement in Table S8 for reproducibility.

^2^
Analyses were also run with the late LPP (1000‐2000 ms). Results are presented in the Supplement on pages 3–4; scalp distribution and waveforms for the late LPP are also included in the Supplement (Figure S4).

^3^
A sensitivity analysis was performed excluding those with less than or equal to 8 segments per condition. Results of these exploratory analyses are provided in the Supplement on pages 2 and 3.
