# Supplementary material for: A Preliminary Examination of Modulation of the LPP by Personal Preference Rankings for Pleasant Experiences
Source: Psychophysiology. 2026 Nov 5;63(11):e70443. doi: 10.1111/psyp.70443 (PMC13740334; doi:10.1111/psyp.70443)
Supplement: Supplementary file 1 [file PSYP-63-e70443-s001.docx]

**Supplemental Results**

***LPP Across all Rankings***

A repeated measures ANOVA across the LPP to all preference rankings 1-8 showed a significant effect of preference rankings (F (7, 504) = 3.43, *p* = .001, *η*^2^*_p_* = .045). LPPs to all positive images were enhanced compared to neutral (*d*s > 0.39, *p*s ≤ .001). The largest LPPs were observed in response to rank 3 (*M* = 3.93, *SD* = 3.88) and rank 5 (*M* = 3.88, *SD* = 3.86). These two conditions did not differ (*d* = 0.01, *p* = .93). The smallest LPP was observed in response to rank 6 (*M* = 3.14, *SD* = 4.07) and rank 8 (*M* = 1.72, *SD* = 3.81). These two conditions differed from each other (*d* = 0.30, *p* = .01) and LPP to rank 8 was reduced compared to all other preference rankings (*d*s > 0.32, *p*s < .01). Together, these findings suggest that although LPP amplitude generally tracks with personal preferences for categories of positive stimuli, it does not map linearly onto each individual ranking and is driven primarily by reduced responding to the lowest-ranked stimuli.

***LPP Modulation by Image Categories***

A repeated measures ANOVA across the LPP to all categories showed a significant effect of category (*F* (7, 497) = 10.68, *p* < .001, *η*^2^*_G_* = .13). All LPPs to positive images were enhanced compared to neutral (*d*s > 0.51, *p*s < .001). The largest LPPs were observed in response to cute animals (*M* = 4.99, *SD* = 4.04) and babies/children (*M* = 4.81, *SD* = 4.09). These two conditions did not differ (*d* = -0.04, *p* = .71), but LPP to cute animals and babies and children were enhanced compared to all other pleasant categories (*d*s > 0.28, *p*s < .05). The smallest LPP was observed in response to cars (*M* = 1.75, *SD* = 3.88) and junk food (*M* = 2.14, *SD* = 3.79). These two conditions did not differ from each other (*d* = 0.09, *p* = .46) but were reduced compared to all other pleasant image categories (*d*s > 0.27, *p*s < .05) except for social activities (*d*s < 0.06*, p*s > .05). As stated above all LPPs to positive images were enhanced compared to neutral, and of particular note, the LPP to cars was significantly larger than neutral, *t*(71) = 3.23, *p* = .002, *d* = .38 and the LPP to junk food was significantly larger than neutral, *t*(71) = 5.54, *p* < .001, *d* = .65. This clarifies that lower-preference categories were not simply functioning as neutral stimuli in the current sample.

***Association of Depressive Symptoms with Rankings, Arousal Ratings, and Valence Ratings***

To examine whether depressive symptoms were associated with rankings and subjective evaluations of the stimuli, correlations were conducted between depressive symptoms and participants' preference rankings, valence ratings, and arousal ratings. Depressive symptoms were not significantly associated with preference rankings, for the preference rankings for any of the categories average valence ratings, or average arousal ratings (*p*s > .05). At the category level, depressive symptoms were positively associated with arousal ratings (such that higher arousal ratings were associated with finding the image less exciting) for baby images, *r* = .23, *p* = .032. Overall, depressive symptoms were not broadly associated with subjective evaluations of the image categories.

***Sensitivity Analyses of LPP Modulation by High vs Low Preference Rankings with Segment Counts*** *≤* ***8.***

As a sensitivity analysis, we repeated the primary analyses after excluding participants with eight or fewer segments contributing to a preference condition. The primary preference effect remained significant, with LPP amplitude significantly enhanced for highly preferred (*M* = 3.68, *SD* = 3.59) compared with less-preferred (*M* = 2.70, *SD* = 3.27) positive stimuli, *t*(62) = 2.77, *p* = .007, *d* = .35. Thus, the primary effect of personal preference on the LPP was robust to excluding participants with relatively few usable segments.

***Modulation of the Late LPP by Highly Preferred Images versus Less Preferred Images***

A repeated measures ANOVA was conducted by entering the late LPP to highly preferred images, the late LPP to less preferred images, and the late LPP to neutral images. The late LPP was significantly modulated by personal preferences (*F*(2, 144) = 29.18, *p* < .001, η²ₚ = .288). To interpret this main effect involving three types of stimuli, follow-up paired-samples *t*-tests were conducted comparing late LPP responses to high-preference and low-preference positive stimuli and to neutral stimuli. Late LPPs to both high-ranked (*d* = 0.84, *p* < .001) and low-ranked (*d* = 0.57, *p* < .001) pleasant objects/experiences were enhanced compared to neutral, and late LPP to high-ranked was enhanced compared to LPP to low-ranked (*d* = 0.31, *p* = .009).

***Secondary Analyses of Depressive Symptoms and Intervention Effects***

Repeated measures ANCOVAs were conducted by entering the late LPP to highly preferred images and late LPP to less preferred images with baseline depressive symptoms or intervention group assignment as a covariate in two separate models.

**Main and Interaction Effects of Depressive Symptoms on the Late LPP Modulation by Personal Preference Ratings:** Results from the repeated measures ANCOVA demonstrated that the main effect of preference was not significant (*F*(1, 70) = 2.38, *p* = .127, η²ₚ = .033), and depressive symptoms did not significantly interact with personal preference rankings (*F*(1, 70) = 0.71, *p* = .402, η²ₚ = .010). No significant main effect of depressive symptoms on the late LPP was found (*F*(1, 70) = 2.07, *p* = .155, η²ₚ = .029).

***Main and Interaction Effects of BPPE on the Late LPP Modulation by Personal Preference Ratings***

No significant group differences (BPPE vs. SS) emerged for the late LPP to high (*t*(71) = 0.04, *p* = .970) and low ranked (*t*(71) = −0.83, *p* = .411) categories.

The repeated measures ANCOVA showed that showed that the main effect of preference remained significant (*F*(1, 71) = 7.45, *p* = .008, η²ₚ = .095), and the BPPE intervention did not significantly change the late LPP by personal preference ratings (*F*(1, 71) = 0.62, *p* = .433, η²ₚ = .009). No significant main effect of BPPE intervention on late LPP was found (*F*(1, 71) = 0.19, *p* = .666, η²ₚ = .003).

**Supplemental Tables**

**Table S1**
Mean Ranking by Category and *Distribution of the Positive Stimuli Ranking % Placed in the Top 3 Categories*

| Category | Mean Ranking | % placed in the top 3 categories |
| --- | --- | --- |
| Cute Animals | 3.68 | 51.1 |
| Babies and Children | 4.61 | 25.0 |
| Nature | 3.78 | 46.7 |
| Sports | 4.69 | 37.5 |
| Adventure | 4.41 | 35.2 |
| Social Activities | 2.35 | 79.5 |
| Junk Food | 5.23 | 19.3 |
| Cars | 7.24 | 5.7 |

**Table S2**

*LPP Segment Counts at Pz by Ranking*

| Ranking | Mean | *SD* | Range |
| --- | --- | --- | --- |
| 1 | 11.33 | 1.26 | 6-12 |
| 2 | 11.42 | 1.1 | 7-12 |
| 3 | 11.26 | 1.23 | 6-12 |
| 4 | 11.45 | 0.85 | 8-12 |
| 5 | 11.4 | 1.18 | 7-12 |
| 6 | 11.25 | 1.05 | 8-12 |
| 7 | 11.36 | 0.86 | 9-12 |
| 8 | 11.22 | 1.24 | 6-12 |
| Neutral | 22.62 | 2.00 | 15-24 |

**Table S3**

*Split-Half Reliability by High and Low Rankings*

| Ranking | Split-Half Reliability |
| --- | --- |
| High Preference (1-3) | 0.79 |
| 1 | 0.63 |
| 2 | 0.64 |
| 3 | 0.58 |
| Low Preference (6-8) | 0.60 |
| 6 | 0.45 |
| 7 | 0.59 |
| 8 | 0.49 |

**Table S4**
Descriptive Statistics for LPP for Each Positive Image Category and Neutral Images Sorted from Largest to Smallest

| Category | *M*_µV_ | *SD*_µV_ |
| --- | --- | --- |
| Cute Animals | 4.99 | 4.04 |
| Babies and Children | 4.81 | 4.09 |
| Nature | 3.47 | 3.82 |
| Sports | 3.32 | 4.25 |
| Adventure | 3.22 | 4.32 |
| Social Activities | 2.31 | 3.99 |
| Junk Food | 2.14 | 3.78 |
| Cars | 1.75 | 3.88 |
| Neutral | 0.19 | 3.52 |

*_Note_*_. LPP = late positive potential._

**Table S5**

*Mean Arousal Ratings by Category*

| Category | *M_arousal_* | *SD_arousal_* |
| --- | --- | --- |
| Cars | 5.71 | 2.09 |
| Social Activities | 4.49 | 1.50 |
| Babies and Children | 4.42 | 1.75 |
| Junk Food | 4.06 | 1.29 |
| Sports | 4.04 | 1.36 |
| Nature | 3.87 | 1.67 |
| Cute Animals | 3.68 | 1.55 |
| Adventure | 3.44 | 1.65 |

*_Note_*_. High arousal ratings on this scale refer to finding the image less exciting._

**Table S6**

*Mean Valence Ratings by Category*

| Category | *M_valence_* | *SD_valence_* |
| --- | --- | --- |
| Cars | 5.04 | 1.18 |
| Sports | 3.85 | 1.08 |
| Adventure | 3.73 | 1.60 |
| Junk Food | 3.43 | 1.20 |
| Social Activities | 3.41 | 1.28 |
| Babies and Children | 2.79 | 1.14 |
| Cute Animals | 2.49 | 1.12 |
| Nature | 2.42 | 0.95 |

*_Note_*_. High valence ratings on this scale refer to finding the image less pleasant._

**Table S7**
*Paired-Samples t-tests Examining the Difference Between Pleasant Images and Neutral Image on Valence and Arousal Ratings*

| Comparison  Pleasant vs. Neutral | *M* (SD) - Pleasant | *M* (SD) - Neutral | Mean diff.  95 % CI | *t* (*df*) | *p* | Cohen’s *d*  95 % CI |
| --- | --- | --- | --- | --- | --- | --- |
| Valence | 3.16(0.73) | 5.70(0.94) | -2.54 [-2.77, -2.30] | -21.34(85) | < .001 | -2.30 [-2.71, -1.90] |
| Arousal | 4.21(1.00) | 6.92(1.40) | -2.70 [-3.00, -2.40] | -17.83 (85) | <.001 | -1.92 [-2.28, -1.56] |

**Table S8.**

*Image Labels and Corresponding Database*

| **Category** | **File Name** | **Description** | **Database** |
| --- | --- | --- | --- |
| *Cute Animals* | | | |
|  | Chipmunk 2 | Chipmunk | Oasis |
|  | Dog 19 | Dog | Oasis |
|  | Dog 5 | Puppy | Oasis |
|  | Cat 5 | Kitten wrapped in towel | Oasis |
|  | Dog 4 | White puppy | Oasis |
|  | Dog 6 | Puppy in a mug | Oasis |
| *Babies and Children* |  |  |  |
|  | Baby 9 | Baby in blue hat | Oasis |
|  | Baby 3 | Baby in pink hat | Oasis |
|  | Picnic 2 | Two girls sharing watermelon | Oasis |
|  | Baby 8 | Two babies in hats | Oasis |
|  | Baby 5 | Baby playing | Oasis |
|  | Siblings 1 | Small child and baby smiling | Oasis |
| *Nature* |  |  |  |
|  | Beach 1 | Sand and ocean | Oasis |
|  | Beach 2 | Ocean sunset | Oasis |
|  | Lake 2 | Lake | Oasis |
|  | Lake 12 | Lake and mountains | Oasis |
|  | Lake 15 | Lake and trees at sunset | Oasis |
|  | Rainbow 2 | Rainbow into lake | Oasis |
| *Sports* |  |  |  |
|  | Snowboarding | Snowboarder | Pexels |
|  | Surfing | Multiple people surfing | Pexels |
|  | Baseball | Baseball players | Pexels |
|  | Kayaking | A person kayaking | Pexels |
|  | Football | Football play | Pexels |
|  | Cyclists | People on bicycles in a race | Pexels |
| *Adventure* |  |  |  |
|  | Parasailing 2 | Person parasailing | Oasis |
|  | Hang gliding 2 | People hang gliding | Oasis |
|  | Skydiving 1 | People skydiving | Oasis |
|  | Parachuting 3 | Person parachuting | Oasis |
|  | Parachuting 2 | Person parachuting | Oasis |
|  | Rafting 4 | White water rafting | Oasis |
| *Social Activities* |  |  |  |
|  | Pos17 | Friends smiling in selfie | IET* |
|  | Pos13 | Friend jumping together | IET* |
|  | Pos20 | Friends smiling white out background | IET* |
|  | Pos18 | Friends smiling in the grass | IET* |
|  | Pos15 | Friends hugging at the beach | IET* |
|  | Pos12 | Two people hugging | IET* |
| *Junk Food* |  |  |  |
|  | Fries 2 | Fries | Pixabay |
|  | Gummy bears | Gummy bears | Pixabay |
|  | Ice cream | Ice cream cones | Pixabay |
|  | Cake | Cake | Pixabay |
|  | Burger | Burger & fries | Pixabay |
|  | Pizza | Pizza | Pexels |
| *Cars* |  |  |  |
|  | Car1 | Indycar | Pexels |
|  | Car2 | Red Nissan | Pexels |
|  | Car7 | BMW M series | Pexels |
|  | Car8 | Alfa Romeo | Pexels |
|  | Car9 | Corvette C5 | Pexels |
|  | Car10 | Aston Martin | Pexels |
| *Neutral* |  |  |  |
|  | Cotton swabs 3 | Cup of cotton swabs | Oasis |
|  | Office supplies 2 | Stapler | Oasis |
|  | Office supplies 1 | Rubber bands | Oasis |
|  | Socks 1 | Pair of socks | Oasis |
|  | Cardboard 1 | Stack of cardboard | Oasis |
|  | Paintbrush 1 | Paintbrush | Oasis |
|  | Keyboard 1 | Computer keyboard | Oasis |
|  | Bottle 1 | Water bottles | Oasis |
|  | Office supplies 4 | Cup of pens | Oasis |
|  | Keys 1 | Pile of keys | Oasis |
|  | Barrels 1 | Couple of barrels | Oasis |
|  | 5740 | Leaf | IAPS |
|  | Timber 1 | Pile of timber | Oasis |
|  | Swingset 1 | Swingset | Oasis |

*_Note._* _*IET = Interpersonal Images Task. Images from this task represent stock photos from a previous interpersonal emotional images task we developed (Dickey, Pegg, and Kujawa, 2021)._

**Supplemental Figures**

**Figure S1**

*ERP Waveforms for LPP to Individual Categories*

**Figure S2**

*ERP Waveforms for LPP to Individual Preference Ratings*

**Figure S3**

*Scatter Plot Depicting the Association between Depressive Symptoms and the LPP Preference Difference Score After Winsorizing the Outlier Value (r* = -.23*, p* = .054*)*

**Figure S4**

*ERP Wave for the Late LPP to Individual Preference Rankings and Scalp Distribution Collapsed Across Neutral, Low Preference Averages, and High Preferences Averages*
